# Early activation of MyD88-mediated autophagy sustains HSV-1 replication in human monocytic THP-1 cells

**DOI:** 10.1038/srep31302

**Published:** 2016-08-11

**Authors:** Gabriel Siracusano, Assunta Venuti, Daniele Lombardo, Antonio Mastino, Audrey Esclatine, Maria Teresa Sciortino

**Affiliations:** 1 Department of Chemical, Biological, Pharmaceutical and Environmental Sciences, University of Messina, 98166 Messina, Italy; 2 Division of Immunology, Transplantation and Infectious Diseases, San Raffaele Scientific Institute, 20127 Milan, Italy; 3 The Institute of Translational Pharmacology, CNR, Via Fosso del Cavaliere 100, I-00133, Rome, Italy; 4 Institute for Integrative Biology of the Cell (I2BC), CEA, CNRS, Univ Paris-Sud, Université Paris-Saclay, 91198 Gif-sur-Yvette cedex, France

## Abstract

Autophagy is a cellular degradation pathway that exerts numerous functions in vital biological processes. Among these, it contributes to both innate and adaptive immunity. On the other hand, pathogens have evolved strategies to manipulate autophagy for their own advantage. By monitoring autophagic markers, we showed that HSV-1 transiently induced autophagosome formation during early times of the infection of monocytic THP-1 cells and human monocytes. Autophagy is induced in THP-1 cells by a mechanism independent of viral gene expression or viral DNA accumulation. We found that the MyD88 signaling pathway is required for HSV-1-mediated autophagy, and it is linked to the toll-like receptor 2 (TLR2). Interestingly, autophagy inhibition by pharmacological modulators or siRNA knockdown impaired viral replication in both THP-1 cells and human monocytes, suggest that the virus exploits the autophagic machinery to its own benefit in these cells. Taken together, these findings indicate that the early autophagic response induced by HSV-1 exerts a proviral role, improving viral production in a semi-permissive model such as THP-1 cells and human monocytes.

Herpes simplex virus type 1 (HSV-1) is a neurotropic α-herpesvirus that infects the majority of human population. It replicates in epithelial cells and establishes latent infections in sensory neurons, causing a variety of clinical syndromes including mild mucocutaneous diseases and life-threatening viral encephalitis. As an obligate intracellular parasite, HSV-1 survival is dependent on its ability to exploit host cell machinery for replication, and to evade intrinsic cellular defences that may limit viral replication, including autophagy^1^.

Macroautophagy (herein referred to as autophagy) is an evolutionary conserved degradation pathway in which cytoplasmic components are sequestered into double membraned structures, known as autophagosomes. Subsequently, autophagosomes fuse with lysosomes to form autolysosomes where the content is degraded by lysosomal enzymes. It is a highly regulated process, in which Beclin1 protein plays a key role in both autophagosome formation and maturation. In addition to its role in development and maintaining cellular homeostasis, autophagy is involved in the innate and adaptive immune responses against pathogens, including viruses, and therefore is considered to be an important antiviral defence mechanism. However, some viruses have evolved strategies to counteract the autophagic response to promote their survival into the host, and also to use the autophagic structures to promote their replication^2^. The current data demonstrate that HSV-1 modulates autophagy by several mechanisms depending on the cell type, leading to different effects on its replication. In particular, HSV-1 counteracts the autophagic response in fibroblasts^3^ and in primary neurons^4^ by the infected cell protein 34.5 (ICP34.5) which directly binds to Beclin1, preventing its functions^3^,^5^. The Us11 protein also inhibits autophagy induction in both HeLa cells and fibroblasts, by direct interaction with the viral sensor PKR^6^. However, autophagy is stimulated in macrophages during the late phases of HSV-1 infection, in order to benefit the host by enhancing the presentation of endogenous viral antigens on MHC class I^7^. In addition, one of the first steps in the immunological response against HSV-1 is the binding of viral components to toll-like receptors (TLRs) which function as pathogen recognition receptors (PRRs). TLRs are transmembrane proteins located either at the plasma membrane or in endosomes. They signal via myeloid differentiation primary response 88 (MyD88) or TIR-domain-containing adapter-inducing interferon-β (TRIF)-dependent pathways, two adaptor proteins recruited to TIR domains upon TLRs stimulation^8^. HSV-1 is recognized by TLR2 and TLR9 and it has been recently reported that the HSV-1-encoded envelope glycoprotein gB is recognized by TLR2, leading to nuclear factor-κB (NF-κB) activation via a signaling pathway involving MyD88 and TNF receptor-associated factor 6 (TRAF6)^9^,^10^,^11^,^12^,^13^,^14^,^15^. Emerging evidences have shown that activation of TLRs can lead to autophagy induction^16^,^17^. Autophagic machinery, stimulated by TLRs signaling pathway, facilitates innate and adaptive immune responses against a variety of pathogens^16^,^17^,^18^.

Monocytes and macrophages are the first lines of defence against viral infections. HSV-1 replicates in these cells, but they are less permissive to viral replication than other cell types. In fact, the virus replicates less efficiently in comparison with fully permissive cell lines, such as epithelial cells. In addition, HSV-1 infects monocytic cells, such as human leukemic monocytic lymphoma (U937) cells or human acute monocytic leukemia (THP-1) cells, with different degrees of permissiveness^19^. Freshly isolated or non-activated monocytic cells are resistant to HSV-1 infection^20^. In contrast, after differentiation to macrophage-like cells, HSV-1 increases the capability to produce infectious particles^20^,^21^, probably representing an important mechanism for virus dissemination^22^. However, the molecular mechanisms regulating the permissiveness of monocytic cells to HSV-1 infection remain poorly understood.

Here, we investigated the relationship between autophagy and HSV-1 replication in THP-1 cells, in order to understand whether autophagy can play a role in the outcome of the infection. We showed that HSV-1 transiently induced autophagy during the early phases of the infection. MyD88 protein is necessary to activate autophagy in HSV-1-infected THP-1 cells. In addition, autophagy stimulation appears to be essential for viral replication in THP-1 cells, suggesting that autophagy plays a proviral role during HSV-1 infection of these cells. Some of the key experiments performed on human monocytes confirm the early activation of autophagy upon HSV-1 infection and its proviral role.

## Results

### HSV-1 transiently induces autophagy during the early phases of infection

**Figure 1:**
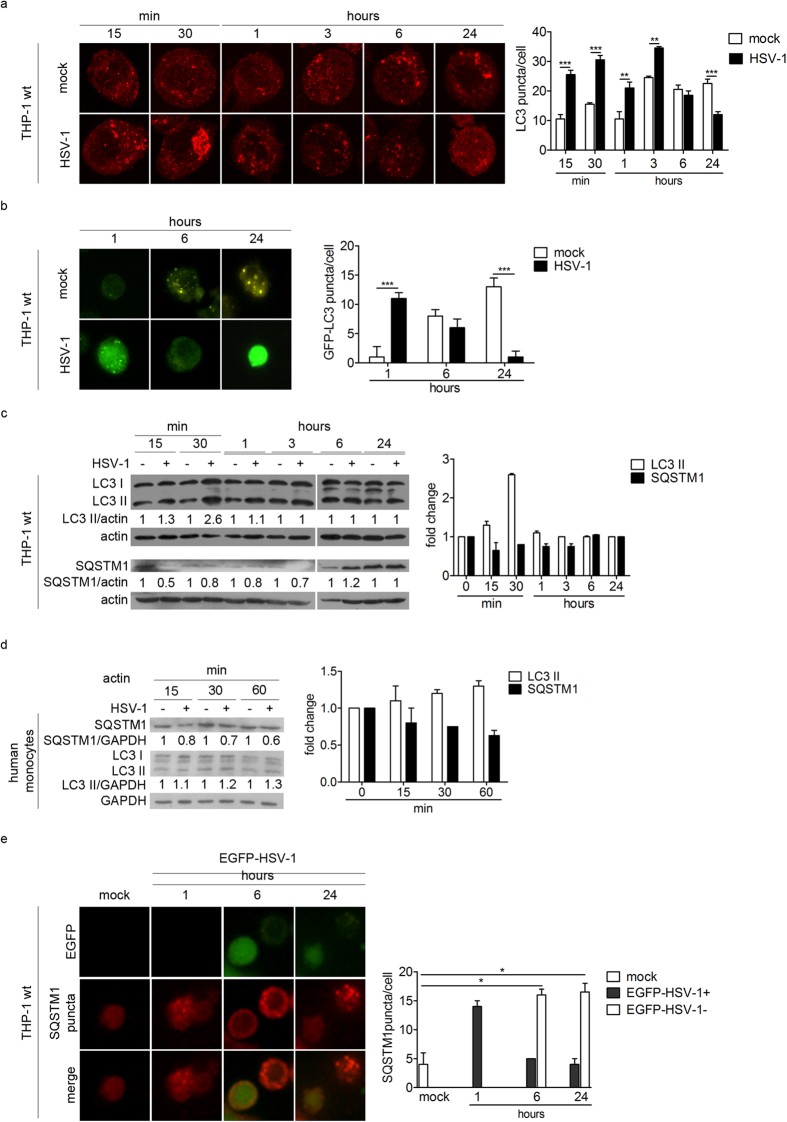
HSV-1 transiently induces autophagy during the early phases of the infection. (**a**) Representative confocal images of THP-1 cells mock-infected or infected with HSV-1, collected at different times and stained for endogenous LC3. Quantification of LC3 puncta was performed using Imaris software; 50 cells were considered for each sample. (**b**) Representative images obtained using an epifluorescence microscope of THP-1 cells transiently transfected with GFP-LC3 plasmid for 6 h, then mock-infected or infected with HSV-1 and fixed at different times. GFP-LC3 puncta were quantified by counting them and 50 cells were considered for each sample. (**c**) Immunoblotting of LC3 and SQSTM1 in THP-1 cells infected or mock-infected at the indicated times. The densitometric analysis of the LC3II/actin and SQSTM1/actin ratios is reported. (**d**) Immunoblotting of LC3 and SQSTM1 in human monocytes infected or mock-infected at the indicated times. The results shown are representative of three independent donors. (**e**) Immunofluorescence images of THP-1 cells infected with EGFP-HSV-1 and stained to identify SQSTM1. Quantification of SQSTM1 puncta in cells expressing EGFP (EGFP-HSV-1 +) or not (EGFP-HSV-1 −). *p < 0.05; **p < 0.01; ***p < 0.001.

We used different assays to assess the impact of HSV-1 infection on autophagy. Autophagy was studied by using two specific markers: LC3, a protein associated with autophagosome membranes, and sequestosome 1 (SQSTM1, also called p62), an autophagic substrate located within autophagosomes. At basal level, the staining of the form I of LC3 (LC3-I) is diffused in the cytoplasm. When an autophagic stimulus occurs, LC3-I is conjugated to the phosphatidylethanolamine (PE) to be converted into form II (LC3-II) which associates with the autophagosomal membrane. First, we infected THP-1 cells with HSV-1 at the multiplicity of infection (MOI) 50, and we evaluated the number of LC3 puncta, corresponding to autophagosomes (Fig. 1a). Confocal analysis showed that infected cells exhibited a greater number of LC3 puncta compared to mock-infected cells from 15 min to 3 h post infection (pi). Conversely, from 6 up to 24 h pi, the number of LC3 puncta was lower in infected cells than in mock-infected cells.

We then transiently transfected THP-1 cells with a GFP-LC3 expression vector and then infected them (Fig. 1b). We observed that infection also increased the number of GFP-LC3 puncta 1 h pi, whereas their numbers strongly decreased at 6 and 24 h pi, confirming the data obtained with endogenous LC3. To note, mock-infected THP-1 cells exhibited a spontaneous increase in the number of autophagosomes at 6 and 24 h, probably related to the stress triggered by nucleofection.

To confirm the transient and early induction of autophagy in HSV-1 infected cells, we also monitored LC3 expression by immunoblot (Fig. 1c). LC3-II was increased after HSV-1 infection and we observed a peak at 30 minutes pi. After 3 h, the level of LC3-II was similar in HSV-1 and mock-infected cells. In addition, we examined the expression of the autophagic substrate SQSTM1. SQSTM1 expression decreased in infected cells during the early times of infection, corresponding to autophagy induction. Further investigations in primary human monocytes from donors confirmed an early activation of autophagy during HSV-1 infection, as indicated by the immunoblot analysis of the two autophagic markers (Fig. 1d).

All together, these data suggest that autophagy was transiently induced after HSV-1 infection both in THP-1 monocytic cells as well as in human monocytes.

In order to know whether the induction of autophagy was limited to HSV-1 infected THP-1 cells or can be triggered in bystander uninfected cells, we constructed a recombinant EGFP-HSV-1 tagged virus (Fig. 1e). In agreement with our previous observations, we observed the accumulation of SQSTM1 puncta, corresponding to autophagosomes, in the cytoplasm of infected cells 1 h pi, but their number decreased at later times in cells expressing EGFP, compared to bystander uninfected cells. This finding suggests that autophagy is stimulated early in cells exposed to the virus, but its inhibition requires that the virus actively replicates.

**Figure 2:**
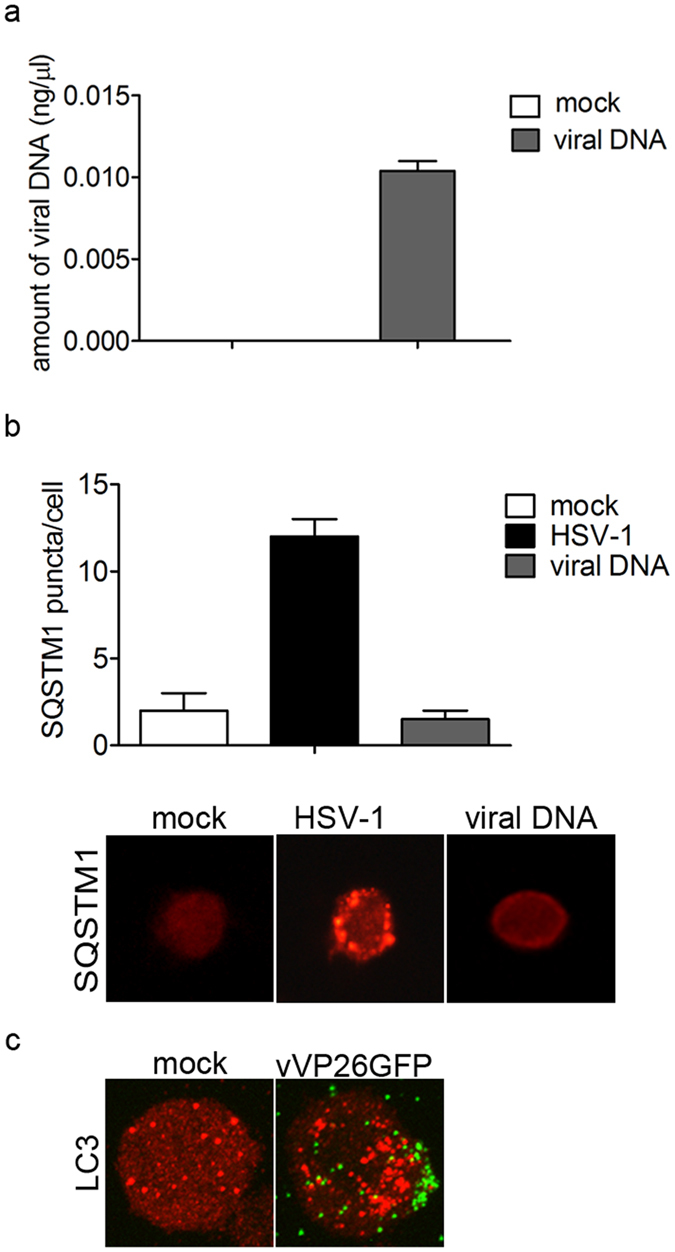
The stimulation of autophagy by HSV-1 is not dependent on viral DNA. THP-1 cells were transiently transfected with viral DNA for 1 h. As a control, transfection of THP-1 cells with GFP-LC3 plasmid and then mock-infected or infected with HSV-1 was used. (**a**) Amount of viral DNA inside THP-1 cells was determined by real-time PCR. (**b**) Representative images of SQSTM1 staining in THP-1 cells transfected with viral DNA. SQSTM1puncta were quantified as previously described. Twenty cells were analysed per assay. (**c**) Representative images of LC3 staining in THP-1 cells mock-infected or infected with VP26GFP HSV-1 for 1 h.

It has been previously reported that HSV-1 was able to induce autophagy in myeloid cells and that viral genomic DNA is essential to trigger autophagy^23^. Based on this, we sought whether HSV-1 genome was involved in the induction of autophagy in THP-1 cells. THP-1 cells were transiently transfected with purified viral genome (BAC-HSV-DNA) and, 1 h after incubation, its presence inside the cells was verified by real time PCR (Fig. 2a). To monitor autophagy, we evaluated the number of SQSTM1 puncta in HSV-1-infected or DNA-transfected cells (Fig. 2b). We did not observe autophagosome formation in cells transfected with HSV-1 DNA. Therefore, we concluded that the early induction of autophagy in HSV-1-infected THP-1 cells was independent of the presence of viral DNA in the cytoplasm. Next, we infected THP-1 cells with a recombinant virus expressing a GFP tagged capsid protein VP26 (vVP26GFP) to visualize incoming viral particles. As shown in Fig. 2c, we observed fluorescent viral particles on the plasma membrane of THP-1 cells 1 h after virus exposure, concomitantly to the stimulation of autophagy. This observation suggests that the early events leading to autophagosome formation in THP-1 cells take place on the cell surface.

### The adaptor protein MyD88 is involved in the early activation of autophagy

**Figure 3:**
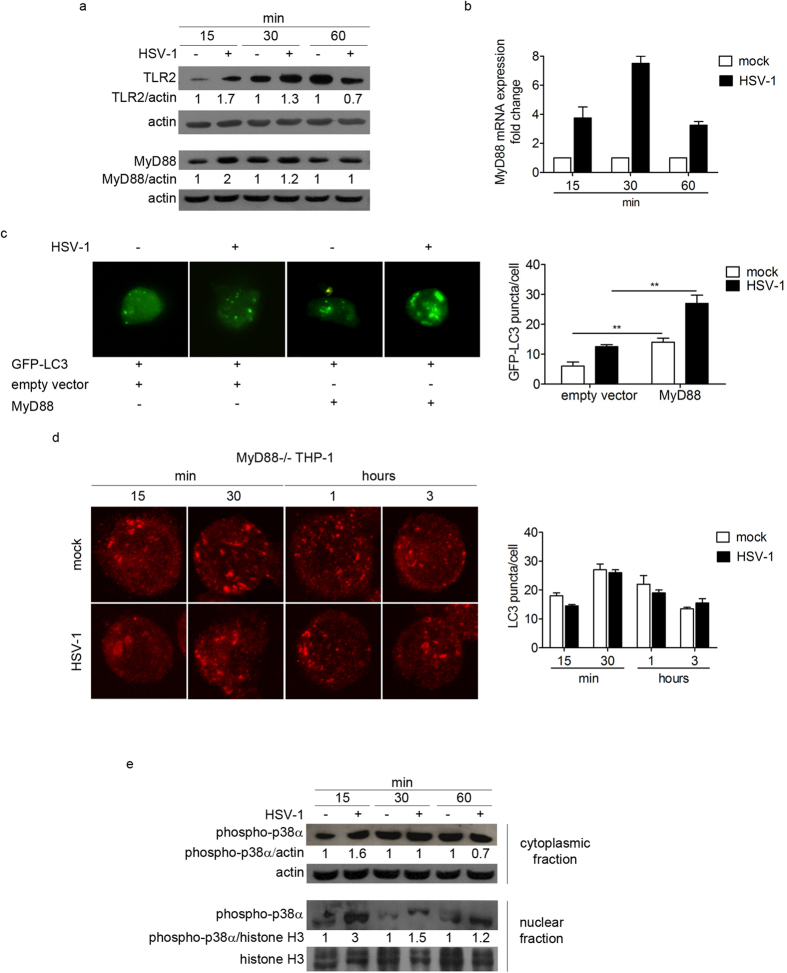
The MyD88-dependent pathway is involved in the early activation of HSV-1-mediated autophagy. (**a**) TLR2 and MyD88 expression of THP-1 cells infected with HSV-1 and harvested at different times after infection. β-actin was used as a loading control. (**b**) MyD88 transcript levels were determined by real-time PCR. (**c**) THP-1 cells were transfected with GFP-LC3 and MyD88 plasmid or pcDNA3.1 as a control and infected with HSV-1 for 1 h. Quantification of GFP-LC3 puncta was performed; 20 cells were analysed for each condition (**p < 0.01). (**d**) Representative confocal images of infected or mock-infected MyD88−/− THP-1 cells stained to identify endogenous LC3 protein. Quantification of LC3 puncta was performed using Imaris software; 50 cells were analysed for each condition. (**e**) Immunoblot analysis of phosphorylated p38α in mock-infected and infected THP-1 cells. β-actin and H3 histone proteins were used as loading controls for the cytoplasmic fraction and the nuclear fraction, respectively.

In order to investigate the mechanism of autophagy induction in THP-1 cells upon HSV-1 infection, we tested the involvement of TLRs-dependent pathway. Indeed, it has been previously reported that TLRs located at the plasma membrane, primarily TLR2, are the first line of defence in the innate response against HSV-1^9^,^13^,^14^,^15^. The immunoblot analysis of TLR2 protein revealed that TLR2 is upregulated in infected cells at 15 and 30 min pi, suggesting a role for this receptor in the early events of HSV-1 infection in THP-1 cells (Fig. 3a). We also monitored the recruitment of the adaptor protein MyD88 as responsible for signal transduction downstream TLRs that are linked to a pathway inducing autophagy^16^,^17^,^18^. First, levels of MyD88 protein and mRNA were evaluated by immunoblot and real time PCR, respectively. MyD88 protein levels were notably increased in infected cells at 15 and 30 min pi (Fig. 3a). In addition, real time PCR analysis of MyD88 mRNA showed that HSV-1 infection induced an accumulation of MyD88 transcripts, with a peak at 30 min pi (Fig. 3b).

As an initial test to investigate MyD88 involvement in autophagosome accumulation, we transiently co-transfected THP-1 cells with MyD88 and GFP-LC3 expression vectors, and infected them at MOI 50 for 1 h. As observed in Fig. 3c, the ectopic expression of MyD88 largely increased the number of GFP-LC3 puncta. Interestingly, the accumulation of GFP-LC3 puncta was higher in the presence of the virus.

To confirm the ability of HSV-1 to trigger autophagy through MyD88 adaptor, THP-1 cells deficient in MyD88 were employed. We observed that HSV-1 infection did not induce autophagy in MyD88−/− cells (Fig. 3d). These results suggest that MyD88 is one of the key factors involved in the signaling pathway leading to autophagy activation mediated by HSV-1 in monocytic cells.

To further investigate the downstream MyD88-dependent signaling pathway in HSV-1 infected cells, we analysed the phosphorylation and subcellular localization of p38α MAPK by immunoblot. HSV-1 induced the phosphorylation of p38α at 15 min pi in the cytoplasm. Conversely, we observed a strong accumulation of the activated form of p38α in the nucleus at 15 min, and a substantial reduction at 30 min and 1 h pi (Fig. 3e). These results suggested that p38α is activated in monocytic cells upon HSV-1 infection and that the early migration of the protein from the cytoplasm to the nucleus could activate downstream events that lead to autophagy regulation.

### Early induction of autophagy exerts a proviral role

**Figure 4:**
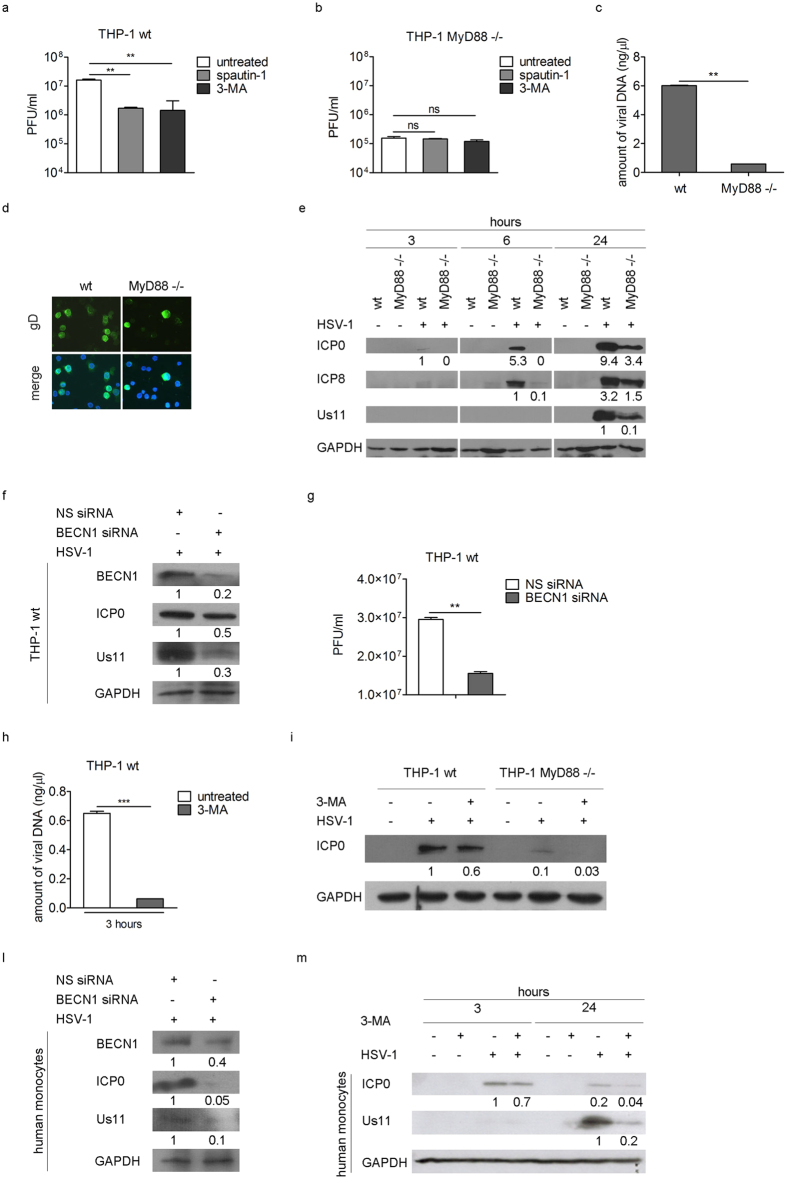
Induction of autophagy mediated by HSV-1 has a proviral role. Viral production was quantified in wt (**p < 0.01) (**a**) and MyD88−/− THP-1 cells (**b**) pre-treated with spautin-1 or 3-MA and infected with HSV-1 in the presence of the inhibitors for 24 h. (**c**) Amount of viral DNA in wt and MyD88−/− THP-1 cells was determined by real time PCR (**p < 0.01). (**d**) Immunofluorescence analysis showing gD-positive wt and MyD88−/− THP-1 cells 24 h pi. Hoechst 33342 was used to stain the nuclei. (**e**) Immunoblot analysis of ICP0, ICP8 and Us11 in wt and MyD88−/− THP-1 cells infected with HSV-1. GAPDH was used as a loading control. (**f**) THP-1 cells were nucleofected with Beclin 1 (BECN1) siRNA for 24 h and then infected with HSV-1. ICP0 and Us11 expression and viral production (**g**) were quantified by immunoblot analysis and plaque assay, respectively. The amount of viral DNA (**h**) was analyzed in THP-1 cells pre-treated with 3-MA and infected with HSV-1 in the presence of the inhibitor for 3 h (***p < 0.001). The same experiment was carried out in wt THP-1 and MyD88−/− cells and ICP0 expression was evaluated by immunoblot (**i**). ICP0 and Us11 expression in human monocytes nucleofected with BECN1 siRNA for 6 h and then infected with HSV-1 for additional 24 h (**l**), or pre-treated with 3-MA and infected with HSV-1 in the presence of the inhibitor for 3 h and 24 h (**m**). The results shown are representative of three independent donors.

With the aim of understanding the function of the early activation of autophagy in THP-1 infected cells, we performed different kinds of analysis. First, we evaluated the effect of autophagy inhibition on virus yield in THP-1 cells by using spautin-1 and 3-methyladenin (3-MA). Spautin-1 is a specific and potent autophagy inhibitor which promotes Beclin1 degradation, by selectively blocking the activity of two ubiquitin-specific peptidases, USP10 and USP13^24^; 3-MA inhibits the class III phosphatidylinositol-3′-kinase (PI3KC3), important to initiate autophagy. THP-1 cells were pre-treated with spautin-1 or 3-MA and then infected with HSV-1 at MOI 50 in the presence of the inhibitor. Twenty four hours later, cells were harvested and viral particle production was quantified by plaque assay on Vero cells. As shown in Fig. 4a, we observed a significant reduction in viral production in cells treated with the two autophagy inhibitors. A similar experiment was carried out with MyD88−/− cells and both drugs had no effect on viral production in the absence of MyD88 (Fig. 4b). However, the data also underlined a different permissiveness between wt and MyD88−/− THP-1 cells (Fig. 4a,b). Indeed, MyD88 deficient cells produce 100 fold less virus than wt THP-1 cells. Similar data were obtained by the real-time quantitative PCR analysis of viral DNA (Fig. 4c) and by the immunofluorescence analysis of HSV-1 infected cells (Fig. 4d). Then, using immunoblot we analyzed the expression of the immediate early protein ICP0, the early protein ICP8, and the late protein Us11 (25) in wt and MyD88−/− infected cells. Figure 4e shows that ICP0 and ICP8 were already expressed in wt THP-1 cells after 6 h of infection, while they were not detectable in MyD88−/− cells. At 24 h pi, ICP0, ICP8 and Us11 expression was markedly reduced in MyD88−/− cells compared to wt THP-1 cells, suggesting a delay of the viral protein accumulation.

In order to verify these results, we then performed siRNA knockdown of Beclin1 (BECN1). We observed that infected THP-1 cells where BECN1 was knocked down showed a reduction of the immediate early protein ICP0 and the late protein Us11, compared to control cells (Fig. 4f). These results were confirmed by the reduction of the viral particle production (Fig. 4g), providing evidence that autophagy inhibition reduces HSV-1 replication. Taken together these results demonstrate that autophagy is essential for the success of HSV-1 infection, since its inhibition affects viral replication. Whether autophagy inhibition would affect the early steps of the infection was evaluated next. THP-1 cells were infected with HSV-1 in the presence or absence of 3-MA and the amount of viral DNA and the expression of the immediate early antigen ICP0 were evaluated. A reduction in the amount of viral DNA 3 h pi was observed in THP-1 cells treated with 3-MA (Fig. 4h) as long as the reduction of ICP0 expression, compared to untreated cells (Fig. 4i). The same experiment was carried out in MyD88−/− cells. As expected, a reduction in ICP0 expression was registered 3 h pi upon pharmacological inhibition, supporting the idea that virus entry is affected upon macroautophagy inhibition. Some hallmark experiments were performed in primary human monocytes. As for THP-1 cells, both the BECN1 knockdown (Fig. 4l) and the 3- MA treatment (Fig. 4m) reduced the expression of the two viral antigens evaluated, ICP0 and US11, in HSV-1 infected monocytes; thus suggests that autophagy plays a positive role in HSV-1 replication cycle in primary cells as well.

### HSV-1 is internalized in THP-1 cells through clathrin-mediated endocytosis

**Figure 5:**
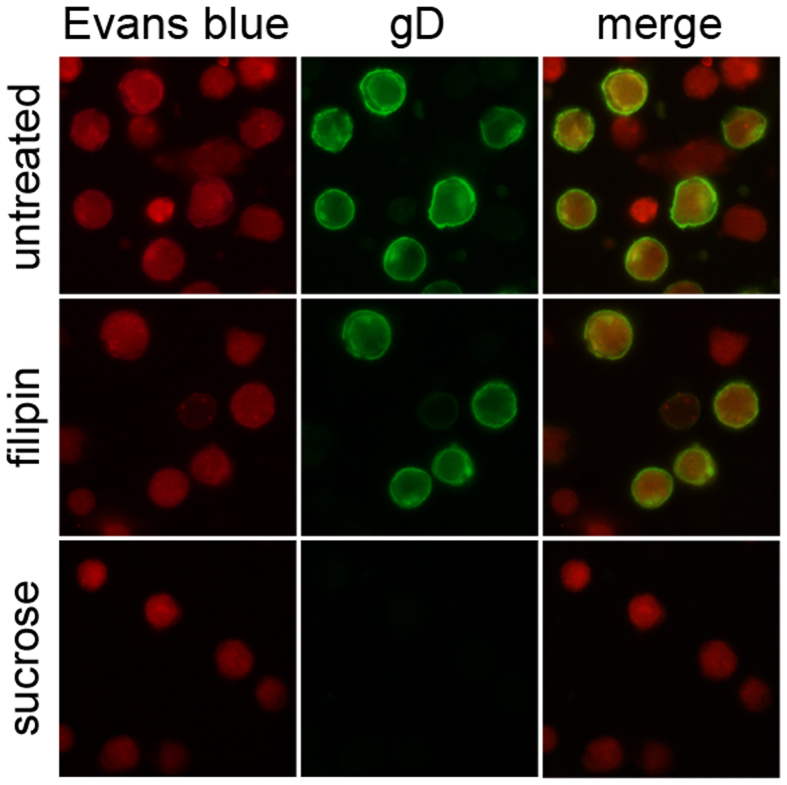
Effect of endocytosis inhibitors on HSV-1 entry. THP-1 cells pre-incubated with filipin or sucrose for 1 h were infected with HSV-1. After 24 h, cells were fixed and stained for gD. Evans Blue dye was used as a counter stain.

Since we observed autophagy stimulation concomitantly with the binding of the virus to the plasma membranes, we finally wondered how HSV-1 is internalized in THP-1 cells. To examine the mode of HSV-1 entry, THP-1 cells were pretreated with non-toxic concentration of two different inhibitors and then infected for 24 h with HSV-1 MOI 50. Hypertonic sucrose medium, a specific inhibitor of clathrin-coated pits pathway, and filipin, a caveolar pathway inhibitor were used. As shown in Fig. 5, whereas filipin had no effect, sucrose treatment inhibited HSV-1 internalization. These results suggest that HSV-1 utilizes clathrin-mediated endocytic pathways to enter into THP-1 cells.

## Discussion

In the present study, we showed that HSV-1 transiently induced autophagy in human monocytic THP-1 cells, independently of the presence of viral DNA or of viral protein synthesis. By using different readouts, we showed that endogenous LC3, GFP-LC3 and SQSTM1 puncta, corresponding to autophagosomes, accumulate in HSV-1 infected cells during the early times of the infection. Conversely, later, autophagy is inhibited or stabilized in cells that allow viral replication, by a mechanism involving neosynthesized viral proteins, probably ICP34.5 and/or Us11, previously reported as anti-autophagic proteins^5^,^6^. As expected, we found that HSV-1 induces autophagy during the early times of its infection in human undifferentiated monocytes as well.

Previously, it has been reported that HSV-1 inhibits autophagy in some cell types^3^,^4^,^5^. However, autophagy is stimulated in murine myeloid cells, and it is not antagonized by ICP34.5^23^, suggesting that the role of ICP34.5 is cell type dependent. In contrast to what was observed in primary neurons^4^,^5^, HSV-1 infection of neuroblastoma cells leads to incomplete or abortive autophagy^26^. We found that in THP-1 cells autophagy stimulation occurred concomitantly with the binding of the virus on the plasma membrane. Indeed, we observed that 1 h after infection, VP26GFP HSV1 particles were bound to the membranes of THP-1 cells. Therefore, we can speculate that the early events that lead to autophagy activation take place on the plasma membrane, probably as a consequence of the binding of viral structural components to cell surface receptors. In support of this, we found that autophagy stimulation is not dependent on the sensing of viral DNA inside the cells, a mechanism observed in myeloid cells^23^. There are several examples of viruses that are able to induce autophagy upon binding to specific receptors on target cells: binding of measles virus to CD46^27^, HIV-1 to CD4^28^,^29^, vesicular stomatitis virus to TLR7 in Drosophila S2 cells^30^,^31^. Innate immune system recognizes the presence of pathogens through pattern recognition receptors, like TLRs^8^. Since HSV-1 is recognized by TLRs^9^,^10^,^11^,^12^,^13^,^14^,^15^, and TLR activation by different ligands induces autophagy^16^,^17^, we explored the TLR signalling pathway during HSV1 infection. We found that TLR2 expression, MyD88 transcription and expression are upregulated early after HSV-1 binding to the cellular membranes. Moreover, ectopic expression of MyD88 induced a significant increase in GFP-LC3 positive autophagosomes in THP-1 cells and, interestingly, this increase was higher in HSV-1 infected cells. Finally, the depletion of MyD88 impaired autophagosomes accumulation in HSV-1 infected cells. All together, these results support a role for MyD88 in the autophagy stimulation by HSV-1 in THP-1 cells. This finding was in line of what has been reported in macrophages, in which the TLR signalling, via its adaptor proteins, leads to autophagy^17^. In addition, we observed that p38α MAPK, a downstream component of the MyD88-dependent signalling pathway, was activated during HSV-1 infection. However, further investigations will be necessary to assess the precise role of p38α in autophagy stimulation.

We next characterized the role of autophagy during the early stages of HSV-1 infection in THP-1 cells. To date, the impact of autophagy on the outcome of HSV-1 infection is controversial, and appears to be cell type dependent^32^. It has been reported that during HSV-1 infection, autophagy limits viral replication *in vitro*^33^, and its virulence *in vivo*^5^,^34^. However, Alexander *et al.* did not observe any impact of autophagy on viral replication in autophagy deficient cells^35^. Interestingly, it has been shown that ICP34.5 inhibits autophagic degradation in dendritic cells, affecting antigen presentation^36^. This could be a strategy used by HSV-1 to evade the host immunity. In addition, in productive HSV-1 infection the virus is able to regulate autophagy by inhibiting or stabilizing the pathway, probably because autophagy may result in viral clearance and reduction of infection^4^,^32^. To study the impact of autophagy on HSV-1 multiplication in THP-1 cells and in human monocytes, we analyzed the pharmacological inhibition of autophagy and the effect of the knockdown of the essential autophagic gene *beclin 1*. Surprisingly, we observed that autophagy inhibition decreased viral production in both THP-1 cells and human monocytes. We observed that in MyD88−/− cells, where no autophagy stimulation is observed, viral production was significantly decreased in comparison to wt THP-1 cells. Moreover, autophagic inhibitors have no effect when MyD88 is lacking. Taken together, these results suggest that autophagy exerts a proviral role in THP-1 infected cells. A proviral role of autophagy has already been shown for other herpesviruses. Recent data have demonstrated that maintenance of the basal levels of autophagy by HSV-2 actively sustains viral replication^37^. Varicella zoster virus induces autophagy, and its inhibition reduces the infectivity, probably impairing viral glycoprotein biosynthesis and processing^38^. Another herpesvirus, Epstein-Barr virus inhibits autophagic degradation and seems to use autophagic machinery for its final envelopment^39^.

Autophagy activation is an early event in HSV-1 infection, therefore, we wanted to analyze the effect of autophagy inhibition 3 h pi, and we found that the amount of viral DNA and the expression of the early antigen ICP0 are already reduced. Since autophagy activation takes place when the virus binds to the cell surface, we can speculate that autophagy may help virus internalization. We then investigated the mode of HSV-1 entry in THP-1 cells and we found that the internalization occurred through the clathrin-mediated endocytic pathway. It is known that there is interplay between autophagic vesicles and endocytotic vesicles^40^. It could be interesting to find out whether autophagy gene products are involved in HSV-1 internalization. The requirement of autophagic machinery for efficient viral entry was already demonstrated for RNA viruses, like Echovirus 7^41^, but no examples of DNA viruses have been reported so far. It has been demonstrated that Atg16L1 interacts with clathrin heavy chains, and preautophagosomal vesicles marked by Atg16L1, Atg5, and Atg12 are formed by the clathrin- and dynamin-dependent uptake of plasma membrane^42^.

In conclusion, we report in this study that the early activation of autophagy upon HSV-1 infection of THP-1 cells and human monocytes have a proviral role, supporting efficient replication, most likely promoting viral internalization. Further investigations are necessary to understand the exact molecular mechanism by which autophagy machinery supports HSV-1 infection in these cells. These studies are important to further elucidate virus-host interactions that could allow the discovery of new molecular targets to design therapeutic approaches against HSV-1 infection.

## Materials and Methods

### Cells and viruses

Wild type THP-1 cells, originally obtained from the American Type Culture Collection (ATCC), were propagated in RPMI 1640 medium (Lonza), supplemented with 10% fetal bovine serum (FBS) (Lonza), glucose (final concentration 4,5 g/L), hepes (10 mM), Lglutamine (20 mM), sodium pyruvate (1 mM), 100 U/ml penicillin and 100 mg/ml streptomycin (Sigma Aldrich). MyD88−/− THP-1 cells from InvivoGen were kindly provided by François Saller (EA4531, Univ. Paris Sud, France). These cells were propagated in the same medium supplemented with zeocin 200 μg/ml and hygromycin 100 μg/ml. Vero cells (ATCC) were propagated in MEM medium supplemented with 6% FBS, 100 U/ml penicillin and 100 mg/ml streptomycin (Sigma Aldrich). All cell lines were maintained at 37 °C in a 5% CO_2_ atmosphere. HSV-1 (F), the prototype HSV-1 strain used in our laboratory, was kindly provided by Pr. Bernard Roizman (University of Chicago, USA). The recombinant EGFP-HSV-1, generated in our laboratory with the aid of the Bacterial Artificial Chromosome, encodes for EGFP tag under the control of α27 promoter cloned in the non coding region spanning between the coding sequences of UL3-UL4 genes (unpublished data). VP26GFP HSV-1 virus (expressing a GFP tagged capsid protein VP26) was a kind gift of Dr. David Pasdeloup (I2BC, Univ Paris Sud, Châtenay-Malabry, France)^43^. Virus stocks were produced and titered in Vero cells.

### HSV-1 infection of THP-1 cells

HSV-1 or EGFP-HSV-1 diluted in RPMI, or RPMI alone (mock-infected) was adsorbed onto cells for 1 h at 37 °C at MOI 50. The inoculum was then diluted with RPMI containing 10% FBS for the indicated times.

### Isolation and infection of human monocytes from peripheral blood mononuclear cells (PBMCs)

Peripheral blood samples were collected from healthy blood donors during routine laboratory analyses at the Blood Bank of San Raffaele Hospital in Milan, according to the rules established by Italian law (Legislative Decree 03-03-2005, published in G.U. n. 85, 13.04.2005). No approval from Ethics Committee was requested because all blood samples were achieved from anonymous donors and the donor could not be identified.

Peripheral blood mononuclear cells were isolated from the buffy coats of three healthy donors by Ficoll-Hypaque density gradient centrifugation. The monocytes were then obtained by Percoll density gradient centrifugation. 80–90% purity was reached, as determined by CD14 expression and other lineage markers, as described elsewhere^44^. The cells were then washed and resuspended in RPMI supplemented with pen/strep (1%), glutamine (1%), heat-inactivated FCS (20%). Monocytes were then seeded at a concentration of 5 × 10^5 ^cells/mL. The day after they were infected with 50 PFU of HSV-1 wt or mock infected as described for THP-1 cells.

### Viral DNA extraction from RR1 bacterial cells

BAC-HSV-1 DNA was extracted from RR1-HSV-1 bacterial cells by using a QIAGEN Maxi extraction kit according to the manufacturer’s instructions^45^.

### Plasmids

The GFP-LC3 expression vector was kindly provided by Tamotsu Yoshimori (Research Institute for Microbial Diseases, Osaka University, Osaka, Japan)^46^. To generate a full-length Myd88 expression-construct, the obtained cDNA was amplified by PCR using a specific primers designed on the basis of the ‘Homo sapiens myeloid differentiation primary response 88 (NCBI GenBank, Accession No. NM_002468.4). The forward primer contained an *EcoRI* site upstream the start codon: EcoRI-forw: 5′CGGAATTCATGGCTGCAGGAGGTCCCGGCGCGG3′ and the reverse primer contained *NotI* site downstream the stop codon: NotI-rev: 5′TTGCGGCCGCTACATGGACAGGCAGACAGATAC3′. The PCR product was double digested with *EcoRI/NotI* enzymes and cloned into the commercial expression vector pcDNA3.1 (Invitrogen).

### Antibodies and reagents

For immunoblot analysis, anti-SQSTM1, anti-LC3 and anti-BECN1 were purchased from BD Biosciences and Sigma Aldrich, respectively. For immunofluorescence studies, anti-LC3 was purchased from Cell Signaling and anti-SQSTM1 from Abnova. Anti-TLR2 was from Genetex and anti-MyD88 was from Cell Signaling. Monoclonal anti- β-actin was provided by Abcam and a polyclonal anti- GAPDH was from Ambion Life Technologies. Antibodies directed against gD, ICP0, phospho-p38α and histone H3 were from Santa Cruz Biotechnology; the antibody against ICP8 was kindly provided by Pr. Bernard Roizman. Horseradish peroxidase anti-rabbit and anti-mouse antibodies were from Santa Cruz Biotechnology. Tetramethyl rhodamine isothiocyanate (TRITC)-conjugated goat anti-rabbit antibody was from Jackson and Alexa Fluor 350 donkey anti-mouse antibody was from Invitrogen. Spautin-1 (10 μM), 3-methyladenin (10 mM), filipin (2.5 μg/ml) and sucrose (0.45 M) were from Sigma Aldrich.

### Immunoblot analysis

THP-1 cells and human monocytes were lysed in 65 mM Tris, pH 6.8, 4% SDS, 1.5% β-mercaptoethanol and held at 100 °C for 5 min. Cytoplasmic and nuclear proteins extraction was carried out as described elsewhere^47^. Gels containing different percentages of SDS-polyacrylamide were used: 15% to resolve LC3 forms I and II, 12.5% for SQSTM1, and 10% for ICP0, ICP8, Us11 and MyD88. After SDS-PAGE, proteins were electrotransferred onto a polyvinylidene difluoride membrane or nitrocellulose membrane. After incubation in blocking buffer, membranes were probed overnight with the specific primary antibody, and then incubated with secondary antibodies, followed by chemioluminescent detection, according to the manufacturer’s instructions (Thermo Scientific). The expression levels of β-actin, GAPDH or histone H3 were used as loading controls. Quantification of protein expression was done by using ImageJ software.

### Immunofluorescence assays

To detect endogenous LC3, cells were layered on polylysinated slides and fixed in methanol for 5 minutes at −20 °C. After blocking and permeabilization with a solution containing 0.2% powdered milk, 2% SVF, 0.1 M glycine and 1% BSA, cells were washed three times with PBS and incubated with an antibody against LC3 for 1 h at room temperature, followed by incubation with the appropriate secondary antibody for 30 min at room temperature. For SQSTM1 and gD immunofluorescence analysis, layered samples on polylysinated slides were fixed in 4% paraformaldehyde for 15 min and permeabilized with 0.1% Triton X-100. To detect SQSTM1, cells were treated with a DAKO blocking buffer, incubated overnight at 4 °C with an antibody against SQSTM1, followed by incubation with the appropriate secondary antibody. To detect HSV-1 gD, cells were incubated with an antibody against gD for 1 h at 37 °C, followed by incubation with the appropriate secondary antibody. Nuclei were stained with Hoechst 33342. Samples were analysed on a Biomed Fluorescence microscope (Letiz, Wetzlar, Germany) or by a Zeiss LSM 510 Meta Confocal Microscope (MIPSIT, Univ Paris-Sud), and Imaris software was used to quantify the number of puncta on confocal images.

### Nucleofection

Cells were transfected using the SG Cell Line 4D-Nucleofector^®^ X Kit (Lonza) in a 4D-Nucleofector™ System, according to the manufacturer’s instructions. After 6 h, cells were infected according to the experimental procedure.

### Knockdown of Beclin 1 by siRNA

Knockdown of Beclin 1 was performed using specific small interfering RNA (sc-29797, Santa Cruz Biotechnology). Briefly, 300 nM siBECN1 or NS-siRNA (5′-AAUUCUCCGAACGUGUCACGU-3′) was used to nucleofect 2 × 10^6^ cells for THP-1 and 2,4 × 10^6^ for human monocytes. The THP-1 cells were then seeded onto 6 well plates for 24 h, whereas the human monocytes cells were seeded onto 24 well plates for 6 h. The cells were then infected according to the experimental procedure.

### RNA extraction, reverse transcription and real time-PCR

Total RNA was extracted using TRIzol^®^ (Life Technologies) according to the manufacturer’s instructions. Total RNA (2.5 μg) was reverse transcribed using avian myeloblastosis virus reverse transcriptase (Promega, Madison, WI). Real-time PCR to detect viral DNA was carried out as described elsewhere^48^. Real time PCR to quantify MyD88 was performed using a Maxima SYBR Green qPCR Master Mix Green real-time PCR with the following primers (Fw-5′GGCATATGCCTGAGCGTTTCG3′; Rev-5′GCGGCACCTCTTTTCGATGAG3′). All samples were tested in triplicates, and mean results were determined.

### Statistics

Data are expressed as a mean ± standard error of the means (SEM) of at least three experiments. One-way analysis of variance (ANOVA) was performed using Prism software (GraphPad).

## Additional Information

**How to cite this article**: Siracusano, G. *et al.* Early activation of MyD88-mediated autophagy sustains HSV-1 replication in human monocytic THP-1 cells. *Sci. Rep.* **6**, 31302; doi: 10.1038/srep31302 (2016).
